# Loss of claudin-3 expression increases colitis risk by promoting Gut Dysbiosis

**DOI:** 10.1080/19490976.2023.2282789

**Published:** 2023-11-27

**Authors:** Rizwan Ahmad, Balawant Kumar, Ishwor Thapa, Geoffrey A. Talmon, Jeffrey Salomon, Amanda E. Ramer-Tait, Dhundy K. Bastola, Punita Dhawan, Amar B. Singh

**Affiliations:** a Department of Biochemistry and Molecular Biology, University of Nebraska Medical Center, Omaha, NE, USA; b School of Interdisciplinary Informatics, College of Information Science & Technology, University of Nebraska at Omaha, Omaha, NE, USA; c Department of Pathology and Microbiology, University of Nebraska Medical Center, Omaha, NE, USA; d Department of Pediatrics, University of Nebraska Medical Center, Omaha, NE, USA; e Department of Food Science and Technology and the Nebraska Food for Health Center, University of Nebraska-Lincoln, Lincoln, NE, USA; f Veterans Affairs Nebraska-Western Iowa Health Care System, Omaha, NE, USA

**Keywords:** Claudin, gut barrier, dysbiosis, FMT, germ-free mice and IBD

## Abstract

Dysregulation of both the gut barrier and microbiota (dysbiosis) promotes susceptibility to and severity of Inflammatory Bowel Diseases (IBD). Leaky gut and dysbiosis often coexist; however, potential interdependence and molecular regulation are not well understood. Robust expression of claudin-3 (CLDN3) characterizes the gut epithelium, and studies have demonstrated a positive association between CLDN3 expression and gut barrier maturity and integrity, including in response to probiotics. However, the exact status and causal role of CLDN3 in IBD and regulation of gut dysbiosis remain unknown. Analysis of mouse and human IBD cohorts helped examine *CLDN3* expression in IBD. The causal role was determined by modeling CLDN3 loss of expression during experimental colitis. 16S sequencing and *in silico* analysis helped examine gut microbiota diversity between *Cldn3*KO and WT mice and potential host metabolic responses. Fecal microbiota transplant (FMT) studies were performed to assess the role of gut dysbiosis in the increased susceptibility of *Cldn3*KO mice to colitis. A significant decrease in CLDN3 expression characterized IBD and CLDN3 loss of expression promoted colitis. 16S sequencing analysis suggested gut microbiota changes in *Cldn3*KO mice that were capable of modulating fatty acid metabolism and oxidative stress response. FMT from naïve *Cldn3*KO mice promoted colitis susceptibility in recipient germ-free mice (GFM) compared with GFM-receiving microbiota from WT mice. Our data demonstrate a critical role of CLDN3 in maintaining normal gut microbiota and inflammatory responses, which can be harnessed to develop novel therapeutic opportunities for patients with IBD.

## Introduction

Gastrointestinal homeostasis depends on the establishment and preservation of gut compartmentalization, in which a single layer of epithelial cells separates the luminal microbiome from the rest of the body. This gastrointestinal epithelial lining forms a permeable barrier that is highly selective and continuously prevents the translocation of luminal antigens to other locations within the body. Tight junctions (TJ), which are intercellular junctions located at the apical end of the epithelial cell layer, are considered critical for regulating the paracellular movement of fluid and solutes and separating the luminal antigens and proinflammatory contents from the body.^1,2^ Altered TJ structure and epithelial permeability have been observed in inflammatory bowel disease (IBD).^3^ Accordingly, impaired gut barrier function is positively associated with gut inflammation, and defects in intestinal epithelial barrier function characterize IBD.^3–5^ However, recent studies have suggested that regulation of tight junction structure and function is rather complex.^3,6,7^ Claudin proteins are integral to both structure and function of TJs; however, they also perform non-canonical functions, as genetic manipulation of claudin proteins in the gut epithelium has resulted in contrasting outcomes.^8–13^ Thus, for therapeutic advantages, it is important to clearly understand the role of specific claudin proteins in gut homeostasis and in promoting IBD.

Claudin-3 (CLDN3) is one of the most highly expressed claudin proteins in the gut and shows a gradient with the highest expression at the crypt top among differentiated colonocytes and the lowest expression at the crypt bottom among poorly differentiated colonocytes.^13^ In addition, CLDN3 serves as a receptor for *Clostridium perfringens* enterotoxin (CPE) and has been investigated extensively for this role.^14,15^ Further studies using IFN-γ, TNF-α, and IL-1β have shown that proinflammatory cytokines downregulate CLDN3 expression in the gut epithelium.^16–18^ A seminal study using a murine model of postnatal gut maturation and probiotic use further showed that CLDN3 expression increases with gut maturation and barrier integrity.^19^ Using *Cldn3*KO mice, we have reported that the loss of CLDN3 expression results in a hyperpermeable gut.^13^ A leaky gut promotes IBD.^3,4^ However, the causal role of CLDN3 in IBD remains unclear.

The gut microbiome plays an important role in maintaining gut homeostasis and in regulating several essential functions, including host metabolism, synthesis of essential vitamins, and protection against pathogens.^20,21^ Dysbiosis is defined as a compositional and functional alteration in the microbiome of individuals with pathological conditions compared to healthy subjects.^20^ A recent report further suggested that gut dysbiosis is not simply a result of inflammation; instead, the dysbiotic microbiome is functionally defective and contributes to inflammation.^22^ Genome-wide association studies (GWAS) further identified links between inflammation and genes involved in microbial signaling, underlining the role of gut microbes in inflammatory gastrointestinal pathogenesis.^23–25^ The clinical benefit of antibiotics or fecal stream diversion also supports the pathological role of bacteria in these disease states.^26,27^ Accumulating evidence suggests that intestinal inflammation alters the microbial community structure.^28–30^ However, the role of gut barrier dysregulation in gut dysbiosis is poorly understood.

In the current study, we show that CLDN3 expression is downregulated to its nadir in IBD patients and murine models of colitis, and the loss of CLDN3 expression promotes the development of colitis and disease severity. We further show that the gut microbiome in *Cldn3*KO mice plays a major role in sensitivity to colitis, emphasizing the therapeutic significance of CLDN3 in promoting intestinal barrier maturity/integrity as well as maintaining normal gut microbiota.

## Results

### CLDN3 expression is markedly downregulated in IBD patients and murine models of colitis

**Figure 1.**
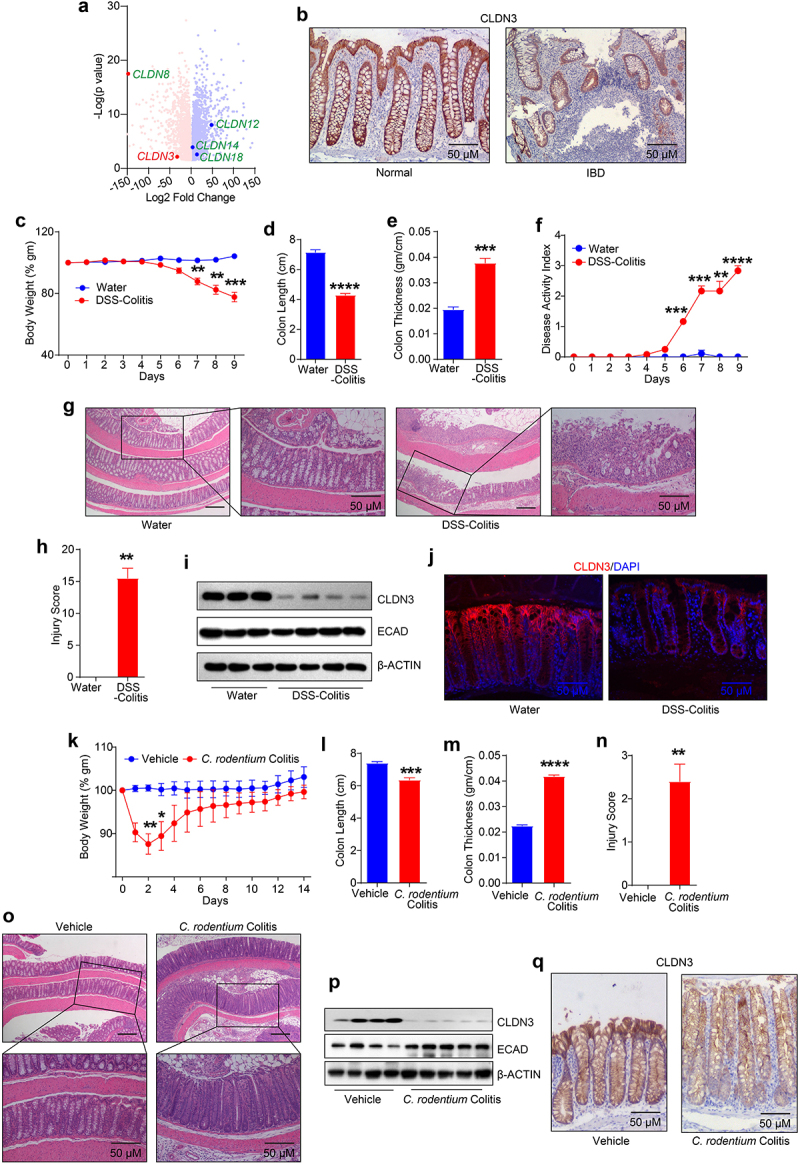
Robust decrease in CLDN3 expression characterize IBD patients and mice subjected to colitis.

To determine the clinical relevance of CLDN3 expression in IBD, we first performed an *in-silico* analysis of *CLDN3* mRNA expression in published IBD patient cohorts (GEO database; see Methods). The results demonstrated a significant downregulation of *CLDN3* transcripts in IBD patients compared to that in healthy individuals (Figure 1a). Immunohistochemical analysis of biopsy specimens from patients with IBD showed a similar downregulation of CLDN3 protein expression (Figure 1b). We next examined status of CLDN3 expression in a murine model of colitis. The mice were subjected to DSS (Dextran Sodium Sulfate) and *C. rodentium* colitis. For DSS-induced colitis, mice administered dextran sulfate sodium (DSS; 2.5% (w/v)) in drinking water were evaluated for colitis-related parameters, including body weight loss, colon length, colon thickness, disease activity index (DAI) and histological grading (by a pathologist) to confirm colitis (Figure 1c–h). For *C. rodentium* colitis, mice received oral gavage of 5 × 10^8^ cfu/100 µl *C. rodentium* (Figure 1k-o). Immunoblot analysis using total tissue lysates from the distal colon showed a sharp decrease in CLDN3 expression in DSS-treated mice compared to that in control mice. E-cadherin (ECAD) expression was not significantly affected in these samples (Figure 1i, j). We found a similar downregulation of CLDN3 expression in the colons of mice subjected *C. rodentium* colitis (Figure 1p, q). Overall, our data established that CLDN3 expression was sharply downregulated in IBD and murine models of colitis.

(a) *In silico* analysis of the published IBD patient (*n* = 360) transcriptomics data demonstrating significantly downregulated *CLDN3* expression *versus* normal (*n* = 153) individuals. ; (b) Immuno-histochemical analysis of CLDN3 expression in biopsy samples from IBD patients; (c) % weight change in mice weight when subjected to acute DSS-colitis (2.5% w/v); (d) Colon length; (e) Increased colon thickness (gm/cm) in DSS colitis; (f) Disease activity index; (g and h) Cumulative injury score and representative images from control and DSS colitis mice; (i) Immunoblot analysis of CLDN3 and ECAD in inflamed and control epithelial cells from colons. β-Actin was used as loading control; (j) Immunofluorescence analysis using anti-CLDN3 antibody in mice subjected to DSS colitis; (k) Change in % body weight in C57B/L6 mice subjected to *C. rodentium* infection; (l) Colon length; (m) Colon thickness in mice subjected to *C. rodentium* colitis; (n and o) Representative H&E images and injury score in mice subjected to *C. rodentium* infection; (p) Immunoblot analysis using anti-CLDN3, -ECAD and β-Actin antibodies infected with *C. rodentium*; and (q) Immunohistochemistry analysis using anti CLDN3 antibody in mice subjected to *C. rodentium* infection. *P < .05, **P < .01, ***P < .001 and ****P < .0001. Scale bar = 50 μm.

### CLDN3 loss-of-expression promotes colitis severity

**Figure 2.**
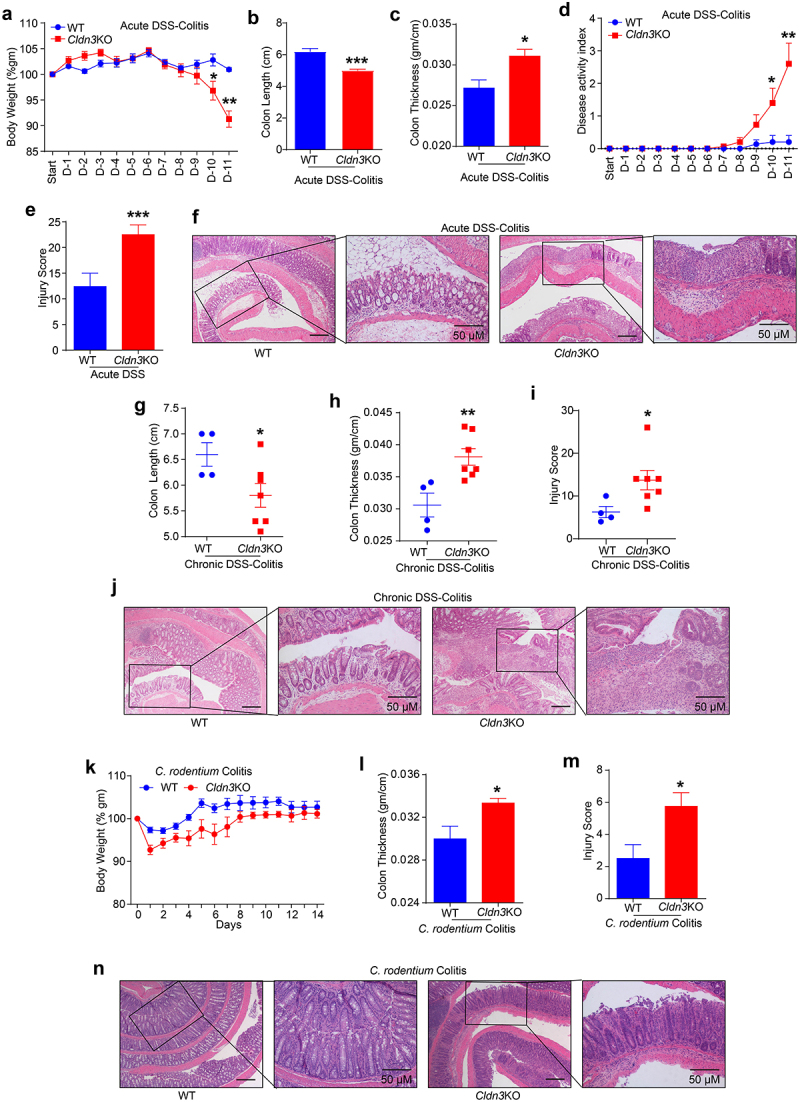
*Cldn3*KO mice are susceptible to colitis.

In light of the above data, we sought to determine whether loss of CLDN3 expression in IBD is simply a bystander effect or has a causal role in IBD pathophysiology. We modeled CLDN3 loss in IBD patients using *Cldn3*KO mice and subjected these mice to DSS in their drinking water. Because of the susceptibility of these mice to higher doses of DSS (data not shown), we used 1% DSS (w/v) over an extended period of 11 days. Compared to WT littermate mice, we found that body weight loss in *Cldn3*KO mice started after day eight and was significantly lower on day-10 and −11 compared to WT-mice (Figure 2a). The colon length and thickness in DSS-treated *Cldn3*KO mice further suggested significant colonic atrophy and edema compared to those in WT mice (Figure 2b,c). DAI and histopathological analysis of the tissue sections of the colon, based on examination of epithelial degeneration, disappearance of crypts, and inflammatory cell infiltration, further revealed that DSS-treated *Cldn3*KO mice had severe mucosal damage compared to WT mice (Figure 2d–f). Considering that IBD is a chronic inflammatory condition, we further examined whether *Cldn3* deficient mice were also susceptible to chronic colitis. For this purpose, we subjected *Cldn3*KO and WT mice to three cycles of DSS administration (5 days/cycle), followed by regular drinking water (16 days/cycle). As shown in Figure 2g, h, significant altrations in colon length and thickness were observed in DSS-treated *Cldn3*KO mice compared to WT mice. The cumulative injury score and histopathology analysis further supported colitis severity in *Cldn3*KO mice compared to WT mice (Figure 2i, j) .

*Cldn3*KO mice and WT littermates were subjected to murine models of colitis to evaluate the role of colonic *CLDN3* in IBD. (a) % change in body weight of *Cldn3*KO mice during acute DSS colitis; (b and c) Colon length and colon thickness in *Cldn3*KO mice versus WT littermates upon acute DSS treatment; (d) Disease activity index; (e and f) Representative H&E images and injury score in *Cldn3*KO mice subjected to acute DSS colitis; (g and h) Colon length and colon thickness in *Cldn3*KO mice subjected to chronic DSS colitis; (i and j) Representative H& E images and injury score in *Cldn3*KO (vs WT) mice subjected to chronic DSS colitis; (k) % change in body weight of *Cldn3*KO mice subjected to *C. rodentium* colitis; (l) Colon thickness in *Cldn3*KO mice versus WT littermates, and (m and n) Representative H&E images and collective injury score in *Cldn3*KO mice compared to WT littermates. *P < .05, **P < .01, and ***P < .001. Scale bar = 50 μm.

However, IBD is a complex disease, and none of the murine models represents the true pathobiology of human diseases. Therefore, we further examined if *Cldn3* deficient mice were also susceptible to *C. rodentium* colitis, which is commonly used to model IBD.^10^ Similar to DSS-colitis, when subjected to *C. rodentium* colitis (for 14 days), significantly altered body weight (% change) and colonic thickness were observed in *Cldn3*KO mice compared to WT mice (Figure 2k, l). To examine whether altered colonic thickness correlated with disease pathobiology, H&E-stained colon sections 14 days post-infection with *C. rodentium* were analyzed. Based on the pathologist scoring of epithelial injury, acute inflammation, chronic inflammation, and depth of inflammation, *Cldn3*KO mice showed significantly higher pathological scores than their WT littermates (Figure 2m, n). Overall, these data affirmed the causal role of CLDN3 in maintaining normal gut homeostasis, and that the loss of CLDN3 promotes colitis.

### CLDN3 loss-of-expression modulates mucosal inflammatory cytokines

**Figure 3.**
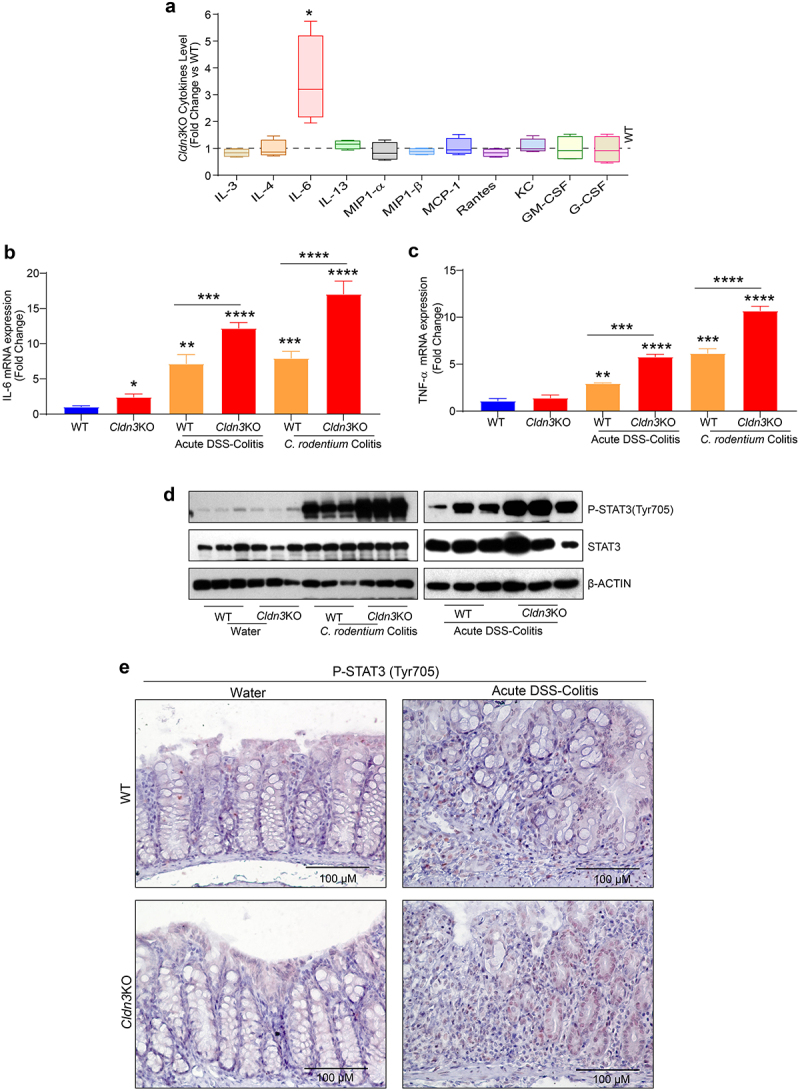
Deletion of *Cldn3* expression exhibits deregulated inflammatory signaling.

We previously reported that the gut in *Cldn3*KO mice is leakier than that in WT mice and shows spontaneous immune cell infiltration.^13^ Evidence suggests that resident infiltrating immune cells in the gut secrete cytokines and chemokines.^31^ Thus, we hypothesized that inflammatory cytokine expression would be dysregulated in *Cldn3*KO mice. Interestingly, a detailed immune analysis using total colon lysates (distal colon) and Multiplex Cytokine ELISA revealed a significant deregulation of basal mucosal immune homeostasis in *Cldn3*KO mice compared to naïve mice. We found that IL-6 levels were significantly upregulated in *Cldn3*KO mice compared to those in WT controls (Figure 3a). There were no changes in IL-3, IL4, MIP-1α/β, IL-13, MCP-1, Rantes, KC, GM-CSF, or G-CSF levels in *Cldn3*KO mice compared to WT mice (Figure 3a). In summary, *Cldn3*KO mice exhibited a specific increase in the expression of IL-6 in their colon.

Multiplex analysis was done using colon from naïve *Cldn3*KO and WT mice. Further, proinflammatory signaling was investigated in *Cldn3*KO subjected to murine models of colitis. (a) *Cldn3*KO mice exhibits dysregulation of cytokine level; (b and c) qRT-PCR analysis for IL-6 and TNF-α cytokines in the colon of *Cldn3*KO mice upon DSS and *C. rodentium* (vs WT); (d) Immunoblots analysis for STAT3 activation in *Cldn3*KO mice upon acute DSS and *C. rodentium* induced colitis; (e) Immunohistochemistry analysis of STAT3 activation in *Cldn3*KO mice compared to WT mice. *P < .05, **P < .01, ***P < .001 and ****P < .0001. Scale bar = 50 μm.

### CLDN3 expression suppresses inflammatory cytokine production and inflammatory signaling pathways during colitis

Considering that IL-6 expression was upregulated in naïve *Cldn3*KO mice, we further examined whether the production of TNF-α, a proinflammatory cytokine targeted in treating IBD, is also altered in *Cldn3*KO mice under conditions of colitis. We measured cytokine levels in colonic tissue harvested from the mice subjected to DSS and *C. rodentium* colitis. Interestingly, upon colitis induction by DSS or *C. rodentium* infection, the loss of CLDN3 expression significantly exacerbated the expression of TNF-α and IL-6 (Figure 3b,c). Considering the persistent increase in IL-6 expression and the fact that STAT3 activation is associated with IBD, we further evaluated the status of STAT3 activation in colon tissues of experimental mice.^32,33^ We examined STAT3 activation by western blot analysis using an antibody specific for STAT3 phosphorylated at Tyr705. As shown in Figure 3d, we found a significant upregulation of STAT3^Y705^ expression in *Cldn3*KO mice compared to WT mice when subjected to colitis. Immunohistochemical analysis to determine the source of activated STAT3 suggested increased activation of STAT3 in both epithelial and immune cells in *Cldn3*KO mice compared to WT mice (Figure 3e). In summary, our data suggest that CLDN3 loss of expression promotes activation of key proinflammatory cytokines and signaling pathways during colitis.

### Cldn3 loss-of-expression promotes susceptibility to colitis in manners dependent on the gut microbiota

**Figure 4.**
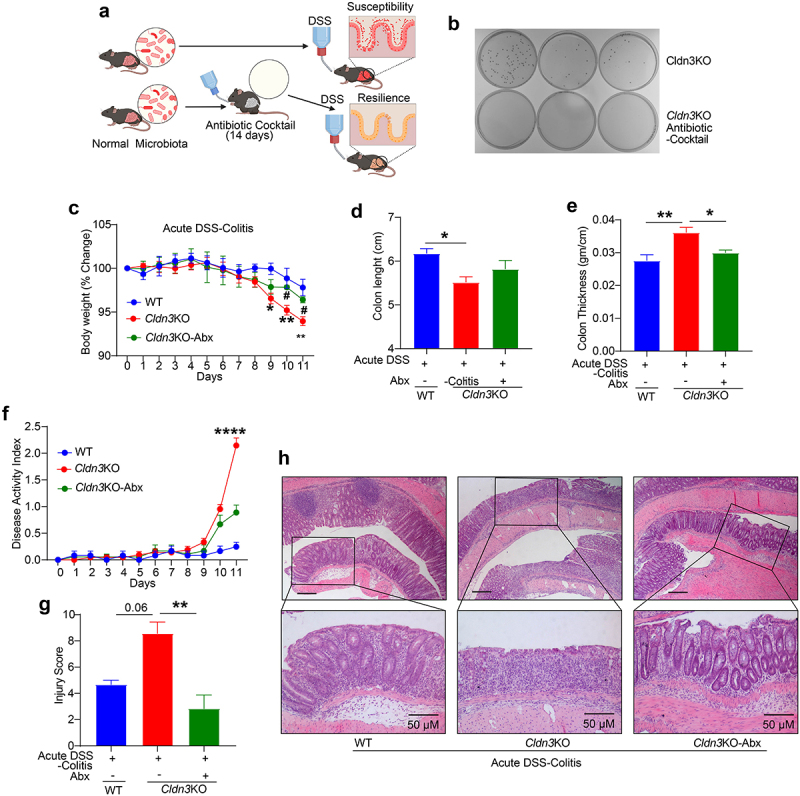
Pseudo-germ-free (microbial depletion) *Cldn3*KO mice show reduced severity of DSS-colitis.

The gut microbiome has emerged as a principal factor in the development and function of the immune system, and published research has linked changes in the gut microbiota to IBD risk and disease severity.^20^ Probiotic treatment promotes CLDN3 expression to promote gut barrier integrity.^19^ Therefore, we examined whether gut microbiome in *Cldn3*KO mice was responsible for their susceptibility to colitis. *Cldn3*KO mice were treated with a broad-spectrum antibiotic (Abx) cocktail in drinking water for 14 days before the induction of experimental colitis to create pseudo-germ-free mice (Figure 4a). As shown in Figure 4b, Abx treatment resulted in robust depletion of the gut microbiota in treated mice. Based on the body weight change and behavior including drinking and eating habits, Abx treatment had no adverse effects on the health of *Cldn3*KO mice. When subjected to DSS-induced colitis, these pseudo-germ-free *Cldn3*KO mice showed striking improvements in body weight loss, colon atrophy or shortening, and edema compared to *Cldn3*KO mice that were subjected to DSS-induced colitis without Abx treatment (Figure 4c–e). DAI and the blinded histological colitis injury scores in Abx-treated *Cldn3*KO mice were also significantly lower than *Cldn3*KO mice without Abx (Figure 4f–h). The histological evaluation and injury scoring based on H&E analysis further showed that the Abx-treated *Cldn3*KO mice had more mucus-filled crypts and reduced inflammatory cell infiltrates compared to *Cldn3*KO mice not treated with Abx (Figure 4h). These findings suggest that susceptibility to DSS colitis in *Cldn3*KO mice is dependent on the gut microbiota.

Broad-spectrum antibiotic cocktail pretreatment was done to evaluate the role of gut microbiota in susceptibility of *Cldn3*KO mice to DSS colitis. (a) Experimental design for the administration of broad-spectrum antibiotics (Abx) and induction of DSS colitis; (b) Fecal microbial culture on LB-agar plate showing microbial depletion in *Cldn3*KO mice upon antibiotic treatment; (c) % body weight of antibiotics treated *Cldn3*KO mice upon DSS treatment compared to non-Abx-treated *Cldn3*KO and WT mice; (d and e) Colon length and thickness in *Cldn3*KO mice exhibits less effects of DSS treatment; (f) Disease activity index, and (g and h) Injury Score and representative H&E analysis reveal a reduction in disease severity in antibiotics treated *Cldn3*KO mice upon DSS colitis compared to non-Abx-treated *Cldn3*KO and WT mice. *P < .05, **P < .01, ***P < .001 and ****P < .0001. Scale bar = 50 μm.

### Cldn3KO mice harbor a more diverse microbial community than WT-mice

**Figure 5.**
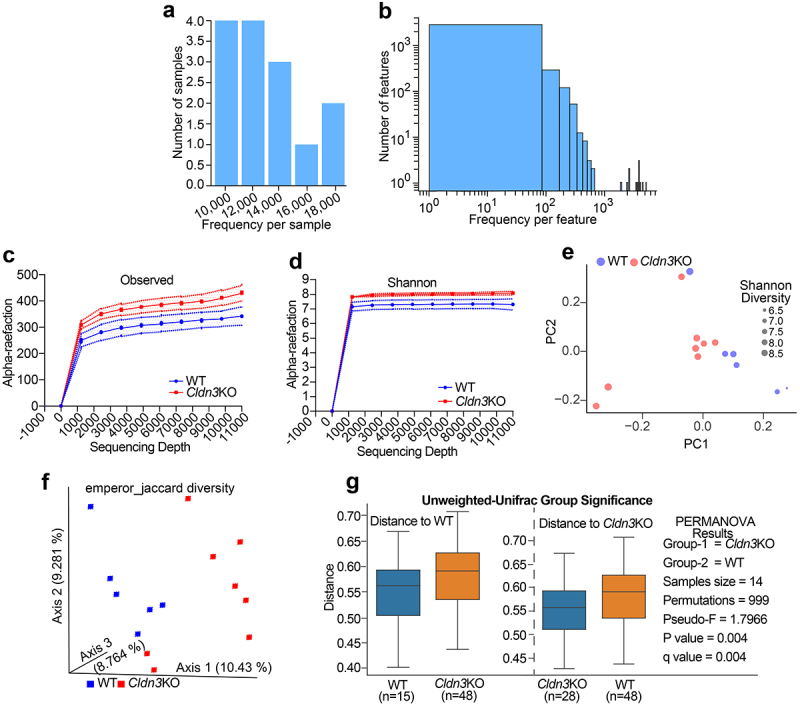
Loss of endogenous *Cldn3* expression alters gut microbial composition.

Based on the above data, the gut microbiome of *Cldn3*KO mice was analyzed to understand how they differ from that of WT mice and the potential mechanisms by which they may affect the host. We collected stool samples from *Cldn3*KO (8 mice) and WT (6 mice) littermate mice and performed 16S rRNA gene sequencing. The resultant data were subjected to a bioinformatics analysis pipeline, which was reflected by the amplicon sequencing variant (ASV) and the frequency distribution in each sample. Figure 5(a,b) show the distribution of the ASVs frequency in each sample and the observed frequency per feature, respectively. Interestingly, the Observed and Shannon rarefaction curves showed less diversity in WT littermates than in *Cldn3*KO mice (Figures 5c,d). Principal coordinate analysis based on Shannon diversity suggested no significant differences between groups (Figure 5e). Jaccard and unweighted-UniFrac β-diversity analyses were performed to compare the gut microbiota community structures across the groups. As shown in Figure 5f, g, we found significant dissimilarities between the microbiomes of *Cldn3*KO and WT samples using Jaccard and unweighted-UniFrac β-diversity analyses. Taken together, these data suggest a distinct microbiome in *Cldn3*KO mice compared to that in WT littermates.

High throughput 16S DNA sequencing profiling analysis of fecal content was carried out using stool genomic DNA from naïve *Cldn3*KO and WT mice. (a and b) The distribution of frequency of features in each sample from *Cldn3*KO mice and WT controls; (c and d) Alpha rarefaction (observed and Shannon) curves show a lower diversity in WT littermates; (e) Principal coordinates analysis showing comparison of alpha diversity (Shannon) in *Cldn3*KO mice versus WT; (f) Principal coordinates analysis showing beta diversity (Jaccard) in *Cldn3*KO mice versus WT, and (g) Unweighted-Unifrac (PERMANOVA) analysis for beta diversity in *Cldn3*KO and WT mice.

### Relative abundance of bacterial communities in Cldn3KO mice differs significantly from that in WT mice

**Figure 6.**
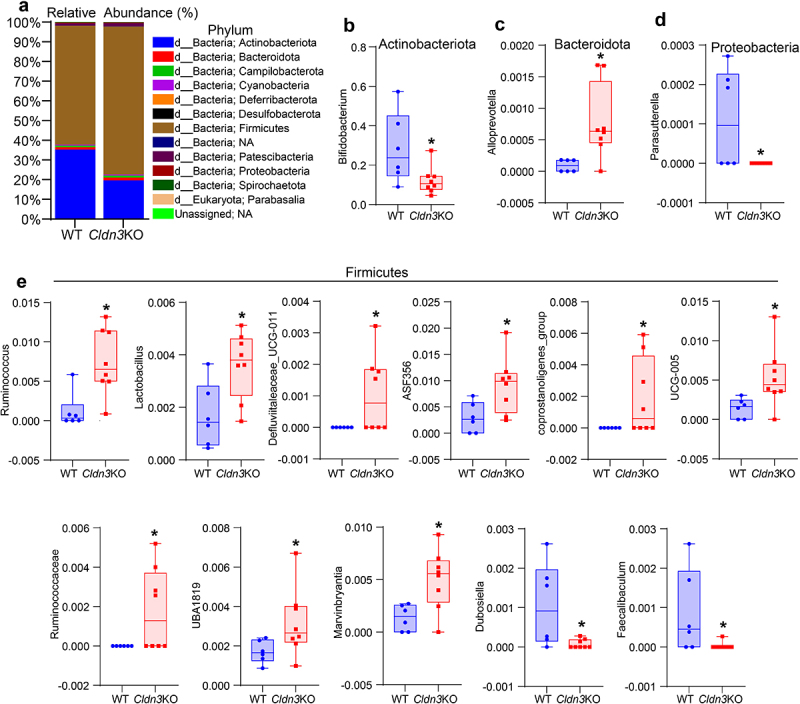
Relative abundance of bacterial communities in *Cldn3*KO differ from WT.

Given that *Cldn3*KO mice exhibit an altered gut microbiota compared to WT animals, we further explored the taxonomic composition of these microbiomes, which showed that Firmicutes (Bacillota) and Actinobacteria (Actinomycetota) were the predominant phyla in all samples from *Cldn3*KO and WT mice (Figure 6a). However, the proportions of taxa from these phyla differed between the two groups. Interestingly, the *Cldn3*KO group, compared to the WT group, exhibited increased levels of the phylum Firmicutes (75.08% vs. 64.41% WT); however, Actinobacteria was reduced from 35.30% (*Cldn3*KO) to 19.65% (vs. WT; Figure 6a). Overall, this observation indicates an altered Firmicutes/Actinobacteria ratio in these mice. We further found ten genera from Bacteroidetes (Bacteriodota) (1) and Firmicutes (9), which were significantly increased in *Cldn3*KO mice compared to WT mice. In contrast, four genera from Firmicutes (2), Proteobacteria (pseudomonadota) (1), and Actinobacteria (1) were lower in *Cldn3*KO mice than in WT (Figure 6b–e). Overall, the above data validated a significant alteration in gut microbiota community profiles in *Cldn3*KO mice compared with WT controls.

Feces were collected for 16S rRNA gene sequencing and QIIME2 software was used to analyze the relative abundance of bacterial communities. Several bacterial taxons were altered with loss of *Cldn3* compared to WT. Wilcoxon rank-sum test of bacterial core genera used in analysis between *Cldn3*KO mice compared to WT. (a) Relative abundance difference at phylum level as shown by the stacked bar; (b) Box plot showing lower abundance of Actinobacteriota in *Cldn3*KO mice (vs WT); (c) Increased Bacteroidota commensal in *Cldn3*KO mice; (d) Proteobacteria was found low in abundance in *Cldn3*KO compared to WT, and (e) Differential abundance of Firmicutes in *Cldn3*KO mice. *P < .05.

### Predicted bacterial functions in Cldn3KO mice implicated significant metabolic changes similar to IBD patients

**Figure 7.**
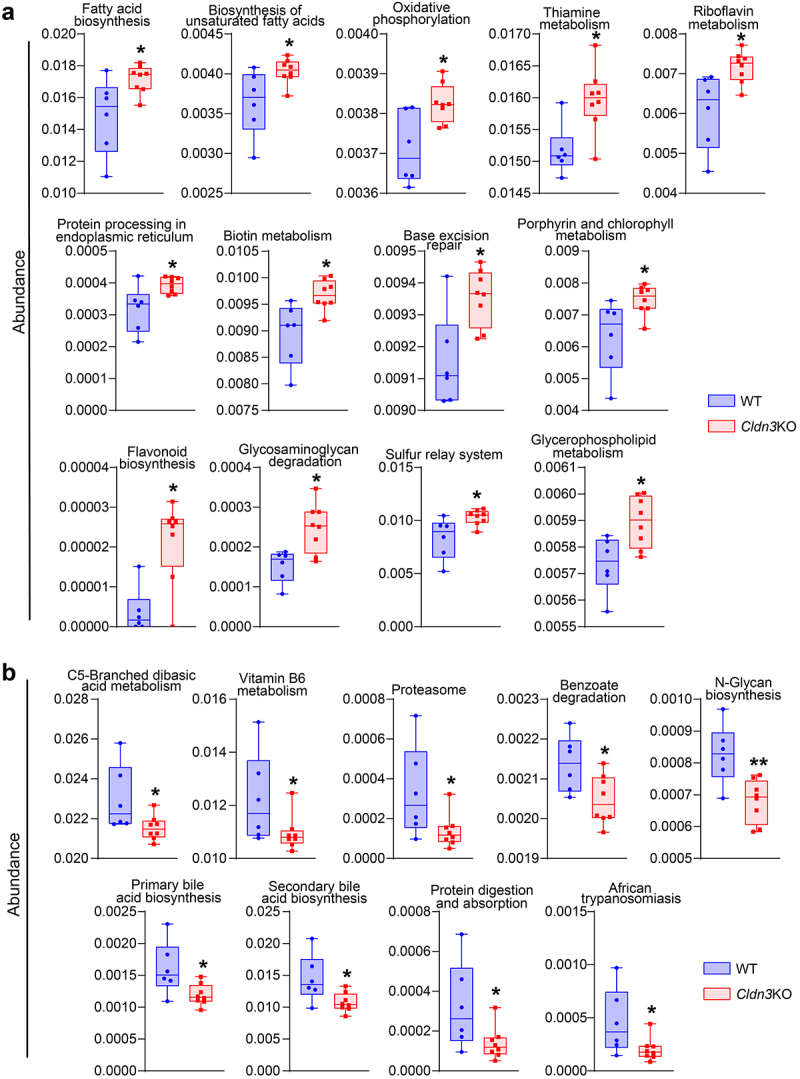
Gut dysbiosis in *Cldn3*KO mice leads to altered host metabolism.

Recent studies have highlighted the significance of microbial metabolites in the causal interplay between microbiome and host homeostasis.^34^ Therefore, to determine how gut dysbiosis in *Cldn3KO* mice may affect their susceptibility to colitis, we determined bacterial gene functions via 16S rRNA gene-based microbial compositions using the R-packages algorithm and the KEGG annotation database. Figure 7a presents the significantly upregulated functional pathways associated with changes in the gut microbiota in *Cldn3*KO mice, including fatty acid metabolism and oxidative phosphorylation, which are known to be altered in IBD patients and promote intestinal inflammation.^35^ The downregulated pathways included the N-glycan, primary, and secondary bile acid biosynthesis pathways compared with WT mice. The metabolism of vitamin B6 and C5-Branched dibasic acid was also downregulated in the microbiomes of *Cldn3*KO mice (Figure 7b). Collectively, these data suggest that predicted alterations in gut microbial functions due to *Cldn3* deficiency could potentially lead to profound changes in bacterial metabolic profiles, which can further affect host metabolism in a manner similar to the metabolic changes in IBD patients.

Based on relative abundance of gut microbiota KEGG functional pathway analysis was done and significant metabolism pathways are presented. (a and b) Up and down regulated KEGG functional metabolic pathways.

### Fecal microbiota transplant (FMT) from Cldn3KO mice to germ-free mice (GFM) promotes colitis

**Figure 8.**
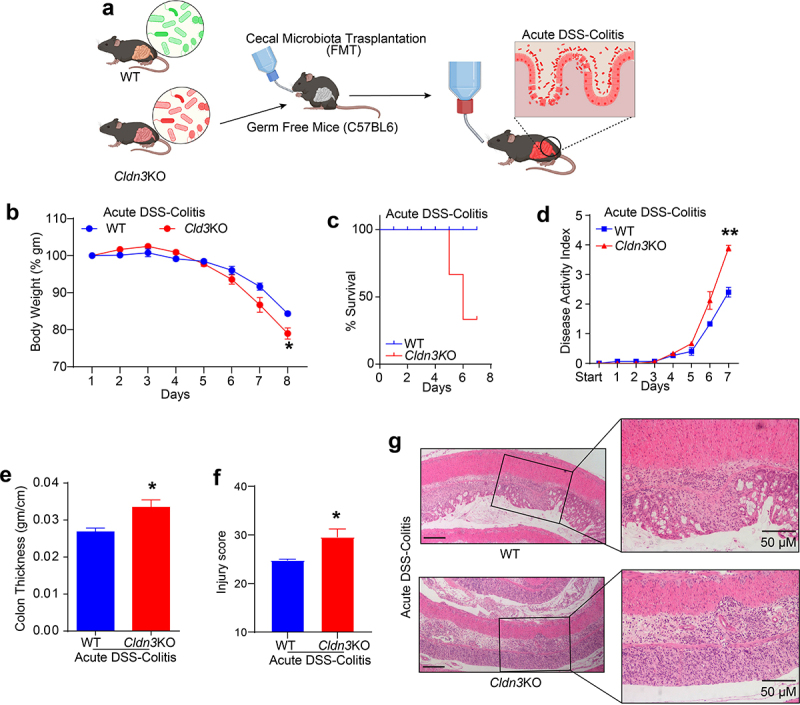
Fecal microbiota transplantation (FMT) from *Cldn3*KO mice to germ-free C57BL/6 mice induces susceptibility to DSS colitis.

Considering that *Cldn3*KO mice are susceptible to colitis and display gut dysbiosis, we hypothesized that the altered microbiome in these mice promotes susceptibility to colitis. To test this hypothesis, we transplanted fecal microbiota from *Cldn3*KO and WT mice into germ-free recipient mice (Figure 8a). After two weeks, no clinical signs of diarrhea or colonic inflammation were noted in the recipient mice. However, when subjected to DSS-induced colitis after the two-week inoculation period, colitis-associated parameters such as body weight loss, overall survival, and disease activity index suggested that recipient mice colonized with the microbiota from *Cldn3*KO mice had severe colitis compared to those colonized with the microbiota from WT mice (Figure 8b–d). We also observed a significant increase in colon edema measured by the weight/length ratio in DSS-treated recipient mice harboring a *Cldn3*KO versus WT microbiota (Figure 8e). Blinded histopathological analysis further supported the severity of inflammation in DSS-treated recipient mice with *Cldn3*KO versus WT mice microbiota (Figure 8f, g). Taken together, our data suggest that the gut microbiota from WT and *Cldn3*KO mice have differential effects on colitis.

Fecal microbiota transplantation (FMT) was performed with germ-free mice to examine the effect of CLDN3 loss mediated dysbiosis on susceptibility to experimental colitis and/or severity. (a) Schematic representation of how FMT and DSS colitis was carried out; (b) % body weight of *Cldn3*KO FMT (*vs* WT-FMT) mice decreases upon DSS treatment; (c) Survival plot showing the death in *Cldn3*KO FMT mice when subjected to DSS colitis; (d) *Cldn3*KO FMT mice showed increased disease activity index compared to WT FMT; (e) Colon thickness increases in *Cldn3*KO FMT mice (vs WT FMT), and (f and g) Representative H&E images and injury score show the susceptibility in *Cldn3*KO FMT mice to DSS colitis *P < .05 and **P < .01. Scale bar = 50 μm.

### Epithelial–microbial interaction drives inflammatory signaling in Cldn3KO mice

**Figure 9.**
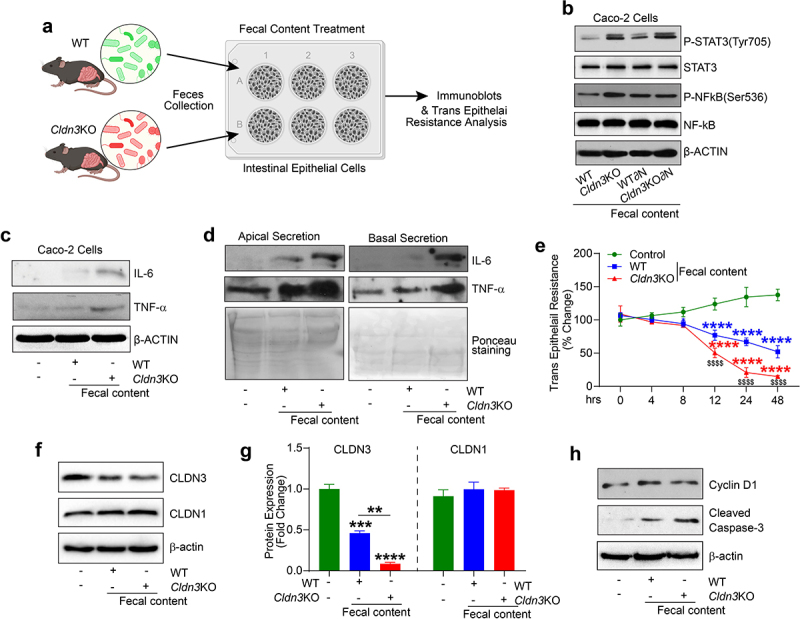
*Cldn3*KO microbiota potentiates inflammatory signaling in intestinal epithelial cells.

To further elucidate the inflammation-promoting potential of dysbiosis in *Cldn3*KO mice, confluent monolayers of Caco-2 cells were exposed to the fecal microbiota content from both *Cldn3*KO and WT mice (Figure 9a). As shown in Figure 9b, phosphorylation of STAT3 (y705) and NF-kB (s536), a measure of proinflammatory signaling, was substantially upregulated in Caco-2 cells exposed to fecal microbiota from *Cldn3*KO mice compared to WT mice. Further analysis for the source of such changes revealed marked upregulation of IL-6 and TNF-α expressions, which were higher in cells exposed to the fecal microbial content from *Cldn3*KO mice compared to WT mice (Figure 9c). We further examined whether observed upregulation of IL-6 and TNF-α were compartmentalized by determining their release in the apical versus basal compartments of the transwell chambers where Caco-2 cells were cultured. As shown in Figure 9d, expression of both TNF-α and IL-6 was higher in the apical compartment than the basal compartment. In light of these findings, we further examined whether exposure of the Caco-2 cells to the gut microbial contents from the WT versus *Cldn3*KO mice affected the barrier integrity. Trans-epithelial resistance (TEER) was measured in control and treated cells over 48 hours. The results using Chopstick Electrode Set (EVOM) showed a significant decrease in TEER in cells treated with the gut microbial content from WT-mice which was further downregulated significantly in cells treated with *Cldn3*KO mice fecal microbiota (Figure 9e). Our data suggest a key role of CLDN3 in gut barrier integrity. Therefore, we wondered if CLDN3 expression was changed in response to the above treatment. Immunoblotting using the total cell lysates showed that CLDN3 expression was indeed downregulated in cells treated with WT-mice fecal microbial content which appeared exacerbated in cells exposed to the *Cldn3*KO mice fecal microbial content (Figure 9f,g). In same lysates, expression of claudin-1, yet other tight junction protein, was not changed. Our additional analysis to examine possible changes in cell proliferation (Cyclin-D1) and death (Cleaved Caspase-3) further showed an increase in the expression of Cleaved Caspase-3 in cells treated with WT-mice microbial contents which was further increased in cells treated with *Cldn3*KO mice microbial contents. Expression of Cyclin-D1 remained largely unaltered (Figure 9h). Overall, our data suggested that fecal microbiota induced proinflammatory signaling, barrier dysfunction and cell death in IECs is potentiated by the fecal microbiota content in *Cldn3*KO mice, similar to colitis in vivo.

*Cldn3*KO and WT fecal microbiota used to determine epithelial-microbial interactions dependent proinflammatory signaling and effect on mucosal barrier function. (a) Schematic representation how experiment was done using fecal microbial content; (b) Immunoblot analysis showing upregulated proinflammatory signaling in *Cldn3*KO fecal content treated cells than WT mice. (c and d) IL-6 and TNF-α production and secretion upon microbial content exposure to caco-2 cells; (e) Trans-epithelial resistance decreases in *Cldn3*KO treated microbial content versus WT; (f and g) Immunoblotting and densitometric analysis for CLDN3, and CLDN1, in Caco-2 cells exposed to fecal content from *Cldn3*KO and WT mice, and (h) Immunoblot analysis using anti-cyclin D1 and -Cleaved Caspase-3 in Caco-2 cells exposed to fecal content from *Cldn3*KO and WT mice. **P < .01, ***P < .001 and, ****, *P* < .0001.

### Signaling pathways and gene expression modulated by an altered gut microbiota show significant association with CLDN3 expression in IBD patients

**Figure 10.**
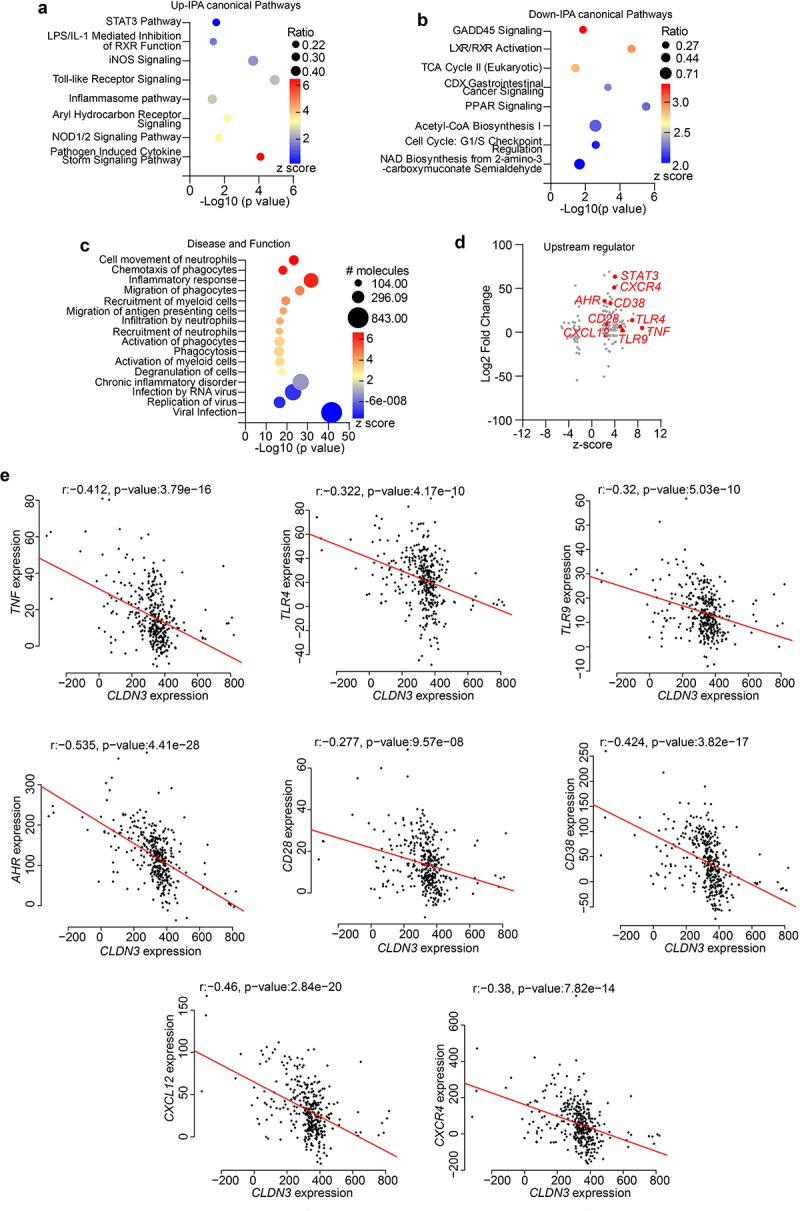
Microbiome associated signaling is altered in IBD and corelates with *CLDN3* expression.

To determine the clinical significance of our findings, we performed an unbiased *in silico* meta-analysis of published high-throughput transcriptomic data from patients with IBD (see Methods). We applied the QIAGEN Ingenuity® Pathway Analysis (IPA®, QIAGEN Redwood City, www.qiagen.com/ingenuity) tool to the DEGs generated using public databases and determined the up- and down-regulated DEGs. In total, 131 significant canonical pathways were identified, among which the upregulated pathways included the STAT3 signaling pathway, LPS/IL-1 Mediated Inhibition of RXR function, iNOS Signaling, Toll-like Receptor Signaling, Inflammasome pathway, aryl hydrocarbon receptor pathway, NOD1/2 signaling pathway, and pathogen-induced cytokine storm signaling pathway (Figure 10a). There were 41 significantly downregulated pathways, including LXR/RXR Activation, TCA Cycle II, CDX Gastrointestinal cancer signaling, PPAR Signaling, and Cell Cycle: G1/S Checkpoint Regulation (Figure 10b). IPA disease and function tool analysis further indicated that bacteria-associated diseases and functions were upregulated in patients with IBD (Figure 10c). The upstream regulator analysis tool is a novel function in IPA analysis that can identify potential upstream regulators, including transcription factors, transmembrane receptors, and any gene or small molecule that has been observed experimentally to affect gene expression, by analyzing the linkage to the DEGs through coordinated expression. *STAT3*, *CD28*, *CD38*, *AHR*, *CXCR12*, *CXCR4*, *TNF*, and *TLR4/9* have also been identified as critical upstream regulators of these genes.^36^ These genes are known to be involved in microbiome-associated signaling pathways, diseases, and functions (Figure 10d).^37^ One of our key findings in IPA upstream regulator analysis was a robust upregulation in host genes that can be affected by the microbiome in the IBD patient dataset (Figure 10d).^37^ In IBD, the expression of upstream regulatory genes is potentially associated with intestinal inflammation and disease severity.^36^ Therefore, we examined the potential association between *CLDN3* and these genes in IBD patients. Using the same patient cohort utilized in the analysis described in Figure 10e, Spearman’s correlation demonstrated a significant inverse correlation between *CLDN3* and *TNF*, *TLR4*, *TLR9*, *AHR*, *CD28*, *CD38*, *CXCR12*, and *CXCR4* expression in the patient cohort. Collectively, our analysis suggests that loss of *CLDN3* modulates microbiome-associated signaling pathways in patients with IBD.

Unbiased functional enrichment of DEGs obtained from in-silico meta-analysis of the previously published high throughput transcriptome from IBD patients was done using QIAGEN’s Ingenuity® Pathway Analysis (IPA) tool. Further interactions between DEGs and dysbiotic microbes were analyzed. (a and b) Canonical up and down regulated ingenuity pathway analysis using GSE database showing enrichment of pathways associated with bacterial signaling in IBD patients; (c) Disease and function analysis; (d) upstream regulator analysis in IBD patient cohorts; (e) Representative plots of Spearman correlation analysis of upstream regulator genes with *CLDN3* expression.

### Gut microbe changes in IBD patients overlap with the microbiome changes in Cldn3KO mice

**Figure 11.**
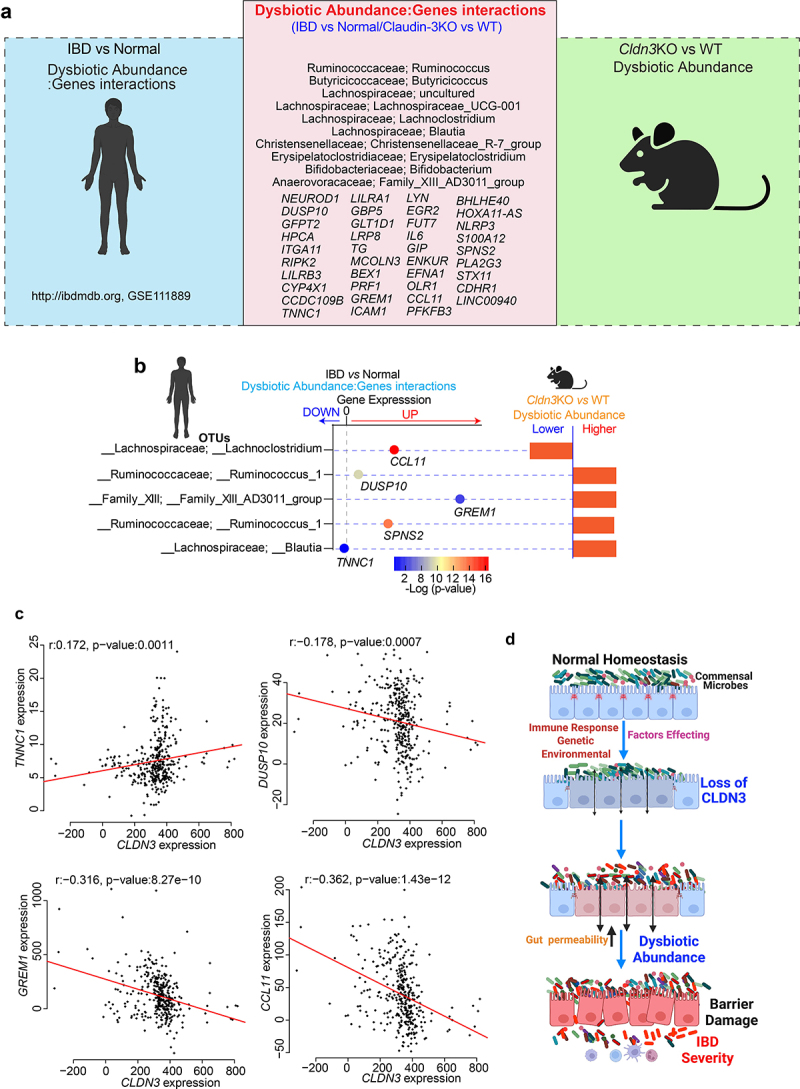
Host-microbiome interactions in IBD patient cohort show overlap with *Cldn3*KO mice.

Having demonstrated that the loss of CLDN3 expression in the mouse gut promotes gut dysbiosis and that these mice have an increased susceptibility to colitis, we further examined the relevance of CLDN3 expression in IBD and associated microbiome changes. To this end, we took advantage of a previously published report in which the authors identified significant gene-taxon pairs in the ileum and rectum of IBD patients.^37^ We compared the dysbiotic taxa from *Cldn3*KO naïve mice (vs. WT littermates) with taxons from a published study.^37^ As shown in a Venn diagram (Figure 11a), we found 10 taxons in naïve *Cldn3*KO mice that aligned positively with dysbiotic taxa in patients with IBD. These dysbiotic taxa included Ruminococcus, Butyricicoccus, Lachnospiraceae_UCG-001, Erysipelatoclostridium, Lachnoclostridium, Blautia, Christensenellaceae_R-7_group, Bifidobacterium, and Family_XIII_AD3011_group (Figure 11a). Further gene/dysbiotic taxon interaction analyses revealed that 39 genes were positively or negatively related to dysbiotic OTUs. Based on the literature, we found genes such *IL-6*, *RIPK2*, *PLA2G3*, *CCL11*, *ICAM1*, and *NLRP3*, which are associated with specific bacterial species present in IBD patients, suggesting that these species are the most susceptible to the activity of these genes.^37^ Further analysis revealed that at least four taxons of IBD patients overlapped with those of *Cldn3*KO mice. These taxons were further associated with critical host genes (Figure 11b). We further examined the potential association between *CLDN3* and these five genes in IBD patients using Spearman’s correlation. Of these five host genes, four showed a significant association with *CLDN3* expression. As shown in Figure 11c, TNNC1 was positively associated with *CLDN3*, *DUSP10*, *GREM1*, and *CCL11* were negatively correlated with *CLDN3*. Overall, these results indicate that the loss of CLDN3 is associated with gut microbial dysbiosis, which promotes inflammation (Figure 11d).

Comparative analysis was performed to determine overlaps in microbial communities between IBD patients and *Cldn3*KO mice, the vital gene interactions with bacteria, and the correlation with *CLDN3* Expression. (a) Representative image showing the overall overlap between host genes: microbiota interaction between human IBD (vs normal) and *Cldn3*KO (vs. WT) mice; (b) Interaction among gene expression in IBD and dysbiotic OTUs from IBD patient and *Cldn3*KO mice; (c) Correlation analysis of genes with *CLDN3* Expression, and (d) Schematic representation of overall concept.

## Discussion

Despite the knowledge that deregulation of gut barrier function and microbiome helps promote susceptibility to intestinal inflammatory diseases, including IBD, and/or disease severity, the causal interdependence between gut barrier dysfunction and dysbiosis remains poorly understood. While frequent co-existence of a leaky gut and microbiota dysbiosis has often been reported, it primarily relates to disease condition(s), and causal relationships are difficult to interpret. In the present study, we used a comprehensive approach that includes microbial profiling in naïve mice, murine modeling of IBD, and *in silico* analysis of the high-throughput data for causal integration between IBD patients and *Cldn3*KO mice. We demonstrate an essential role for CLDN3 in maintaining the normal microbiome. To the best of our knowledge, this is the first report to demonstrate that the loss of CLDN3 expression is sufficient to induce alterations in the gut microbiota that promote susceptibility to IBD as determined by the FMT studies. We previously reported that CLDN3 loss of expression induces gut permeability in mice.^13^ Taken together, CLDN3 appears to be the link connecting the gut barrier and the microbiota. This postulation is supported by a published report that CLDN3 expression is positively associated with the maturity of the gut barrier^19^. Moreover, beneficial bacteria (probiotics) promote gut barrier integrity by specifically promoting CLDN3 expression.^19^ Other studies have shown similar positive association of CLDN3 expression with gut barrier integrity.^38^ These data, when combined with the fact that CLDN3 expression is markedly suppressed in IBD patients and murine models of colitis, emphasize the significance of promoting CLDN3 in improving the prognosis of IBD.

Importantly, our data showing that CLDN3 loss-of-expression not only promotes gut permeability but also induces gut dysbiosis is significant, as regulation of the gut barrier structure/function is complex.^3,6,7^ The claudin family of proteins is now accepted as the principal regulator of tight junctions, and the dysregulated expression of multiple claudin proteins has been reported in IBD.^39,40^ However, related studies, especially those using mice genetically manipulated for specific claudin proteins, have identified non-canonical roles for specific claudin proteins and are possible contributors to intestinal pathobiology. Overexpression of claudin-1 protein in the gut epithelium, to model claudin-1 expression in IBD, promotes colitis by primarily affecting IECs differentiation in Notch- and Wnt-dependent manners.^41^ Similar murine modeling of claudin-2, the leaky claudin, in mice to determine a causal role in IBD, has presented diverse outcomes depending on the colitis model, which suggests complex roles of claudin-2 in intestinal homeostasis.^8,10,11,42^ While the status of claudin-4 in IBD is not clear, based on a recent preliminary report, it appears that claudin-4 upregulation may exacerbate colitis by regulating epithelial injury/repair.^43^ Similarly, results in mice genetically manipulated for claudin-7, yet another high expression of claudin protein in the gut suggests a critical role for claudin-7 in regulating cell-extracellular matrix interactions and stem cell functions.^44^ Of note, claudin-7 KO mice also demonstrate a dysbiotic gut microbiota, but only after being subjected to DSS-colitis.^44^ Claudin-15, another claudin protein expressed in the intestine at high levels, is also reported to be downregulated in IBD.^45^ However, simple genetic manipulation of claudin-15 leads to a mega-intestine phenotype, suggesting that this protein also manifests functions other than tight junctions in the gut.^46^ Taken together, our data show that CLDN3 loss not only induces a leaky gut phenotype but also promotes gut dysbiosis and susceptibility to IBD, suggesting that its targeting (to promote) can not only correct the gut barrier integrity but also improve dysbiosis and colitis risk. Our previously published report that CLDN3 loss of expression promotes the risk of colitis-associated cancer (CAC) further supports this postulation.^13^ However, CLDN3 loss also affects the normal homeostasis of other vital organs, including the liver and bile acid secretion.^47^ Changes in bile acid levels are associated with gut inflammation and colitis.^37,48–50^ Thus, further studies are needed to delineate the role of the gut epithelium specific changes in CLDN3 expression upon gut dysbiosis and colitis and are part of our ongoing studies.

Although the precise etiology of IBD remains unclear, it is thought that the altered immune homeostasis characterizing IBD can result from crosstalk among genetic susceptibility, barrier dysregulation, and gut microbiota, potentially leading to gut dysbiosis, thus allowing the growth of pathogenic microorganisms.^25,37,51,52^ Accordingly, gut dysbiosis in IBD is characterized by a decrease in alpha diversity.^53^ We observed a decrease in alpha diversity in *Cldn3*KO mice. In addition, Jaccard and unweighted-UniFrac β-diversity analyses showed dissimilarities between the gut microbiome of *Cldn3*KO mice and WT littermates.^37,52^ Specifically, the predominant changes were altered ratios of four major phyla, Firmicutes, Actinobacteria, Bacteroidetes, and Proteobacteria, in *Cldn3*KO mice compared to WT mice. Notably, *Bifidobacterium* increases with gut maturation and produces short-chain fatty acids (SCFA). *Bifidobacterium* administration significantly alters the abundance of *Lactobacillus* and contributes to the remission of intestinal inflammation and injury by constituting a favorable gut ecosystem, possibly modulating regulatory T cells and the SCFA environment.^54^ Additionally, *Bifidobacterium* levels are reduced in the gut of IBD patients.^55^ Most notably, similar to IBD patients, we observed a markedly low abundance of *Bifidobacterium* in *Cldn3*KO mice.^55,56^ Despite increased colitis susceptibility in *Cldn3*KO mice, our data demonstrated an unexpected increase in the beneficial phylum Firmicutes in *Cldn3*KO mice guts; however, the overall diversity of the bacterial communities in this phylum was altered. Specifically, we found a higher abundance of proinflammatory genera such as *Ruminococcus* and *Marvinbryantia* in *Cldn3*KO mice, which are known to be associated with gut inflammation and bowel dysfunction.^57–59^ Recent studies using mouse modeling of colitis and associated colon cancer showed a similar reduced abundance of Firmicutes organisms, such as *Dubosiella* and *Faecalibaculum* as found in *Cldn3*KO mice, supporting a colitis-promoting environment in *Cldn3*KO mice.^60,61^ In this regard, a recent study elegantly showed that the autochthonous murine microbiota member *Faecalibaculum rodentium* and its human homolog *Holdemanella biformis* protect against intestinal tumor growth.^62^ Overall, these data support the possible role of colitis-promoting gut microbiota in the higher risks of colitis in *Cldn3*KO mice. However, further investigations using these altered microbes are warranted to outline the role of intestinal CLDN3 expression in gut dysbiosis and IBD.

Importantly, dysbiosis promotes a shift in intestinal homeostasis toward a proinflammatory environment and thus increases the expression of proinflammatory mediators and response gene expression.^20,63^ In this context, our analysis of the published transcriptomic data from IBD patients in relation to gut microbiome revealed STAT3, iNOS, Toll-like receptor, inflammasome, and NOD1/2 signaling pathways as upregulated pathways. We also found increased expression of IL-6 and STAT3 signaling in *Cldn3*KO mice with colitis. In this regard, *Butyricicoccus*, associated with higher IL-6 levels in IBD patients, showed overlapping presence between IBD patients and *Cldn3*KO mice.^37^ On the other hand, *Bifidobacterium* and *Dubosiella* which are low in *Cldn3*KO mice associate negatively with IL-6, STAT3, and NFkB expression.^64,65^ We speculate that these dysbiotic microbiota under condition of *Cldn3* loss promote pro-inflammatory signaling. In support, exposure of polarized IECs to the microbial contents from WT mice induced expression of IL-6 and TNF-α which was exacerbated by gut microbial contents from *Cldn3*KO mice. Notably, NFkB can promote secretion of TNF-α, and IL-6; however, high IL-6 levels can also trigger the NFkB-dependent release of TNF-α, which can result in a feedback loop to aggravate inflammation.^66,67^ We have reported upregulation of gp130, the IL-6 receptor, in *Cldn3*KO mice and CLDN3 knockdown Caco-2 cells.^13^ Taken together, our data presents two plausible scenarios: 1) microbiota-dependent activation of an autocrine IL-6/gp130/STAT3 signaling under low CLDN3 conditions; and 2) microbiota-mediated upregulation of IL-6 in IECs diffuses basally to activate the immune cells, triggering proinflammatory signaling pathways, including STAT3 activation and NFkB, which then aggravate gut inflammation by feedback mechanism. Published studies support compartmentalized and context-dependent activation of the IL-6/STAT3 signaling during inflammation.^68,69^ However, additional studies are needed to mechanically verify above postulations and part of our ongoing studies.

Next, we examined the potential association between *CLDN3* and these genes in IBD patients. Notably, when we examined the same IBD patient cohort used in the above analysis, Spearman’s correlation demonstrated a significant inverse correlation between *CLDN3* and *TNF*, *TLR4*, *TLR9*, *AHR*, *CD28*, *CD38*, *CXCR12*, and *CXCR4* expression. Collectively, our analysis supported the postulation that the loss of *CLDN3* can modulate microbiome-associated signaling pathways in IBD patients and thus promote disease severity.

However, to increase the risk of IBD and/or disease severity, it is critical that host metabolism is affected by genetic or environmental factors. In this context, gut microbiota plays a critical role in regulating host metabolism.^34^ Accordingly, our analysis using the R-package algorithm for microbial abundance based on KEGG annotations revealed that the gut microbiome of *Cldn3*KO mice had the potential to significantly upregulate functional pathways, including fatty acid metabolism and oxidative phosphorylation. The downregulated pathways included the N-glycan, primary, and secondary bile acid biosynthesis pathways compared to WT mice. The metabolism of vitamin B6 and C5-Branched dibasic acid was also downregulated in *Cldn3*KO mice. In this regard, an interplay between the microbiome and systemic and fecal metabolic environments has recently been described.^34^ Collectively, these data suggest that alterations in the gut microbiota due to *Cldn3* deficiency lead to changes in the metabolic profiles of the mouse gut.

Overall, our data in the current study provide strong evidence for a critical role for CLDN3 protein in regulating gut barrier functions and microbiota composition. Further analysis using IBD patient cohorts and murine models of colitis provides additional evidence for the causal role of CLDN3 loss in promoting IBD. Considering that *Cldn3*KO mice present both phenotypes, leaky gut and microbial dysbiosis, it remains unanswered whether increased gut permeability precedes gut dysbiosis or vice versa. Irrespective, these data provide novel information regarding the regulation of gut leakiness and microbiota homeostasis in IBD and its future clinical relevance.

## Materials and Methods

### IBD patient samples

All work in this study was carried out under the Institutional Review Board approved for Clinical Research at the University of Nebraska Medical Center (UNMC), Omaha Nebraska, USA. All deidentified IBD patient sample slides were obtained from archived tissue cores and confirmed by a pathologist by histological examination.

### Cldn3*KO mouse*

All mice used in this study had a C57BL/6J background. The generation of constitutive *Cldn3*KO mouse has been described.^13^ All animal experiments were performed according to institutional guidelines. All mice were kept in individually ventilated cages in compliance with the Animal Welfare Act to avoid microbial cross-contamination with *Cldn3*KO and WT mice. Mice were housed under a 12-hr day and night cycle with free access to food and water. *Cldn3*KO mice were bred with their WT littermates. After genotyping, the mice were separated into two groups and maintained for at least four weeks to ensure that microbiota contamination was not an issue.

### Models of experimental colitis

All mouse studies were approved by the UNMC Animal Care and Use Committee. All mice were provided a regular chow diet ad libitum. Matched *Cldn3*KO and WT littermate mice (8–10 weeks old male and female) were used. The experimental colitis models used in this study are described below:

#### Acute and chronic DSS colitis

Male and female mice from *Cldn3*KO and WT littermates were grouped randomly into respective groups, and colitis was induced by dextran sodium sulfate (DSS; 40 kDa; TdB Labs, Sweden) as described previously.^8^

#### C. rodentium *induced colitis*

*C. rodentium* strain DBS100 (ATCC 51,459; USA) was grown by shaking overnight in Luria-Bertani broth at 37°C. Mice were infected by oral gavage using 0.1 ml of Luria broth containing 5 × 10^8^ cfu of *C. rodentium*. To minimize the differences between infections, both *Cldn3*KO and WT littermate mice were infected with the same bacterial preparation. The mice were monitored for clinical symptoms and signs of colitis, and their body weight was recorded every day. On day 14 following infection, mice were euthanized, and colon tissues were prepared for histological, protein, and RNA analyses.

#### Evaluation of colonic thickness/edema

The colonic weight/length ratio is an indirect method to determine the colon thickness or edema in the murine models of experimental colitis, which correlates well with the histological score.^8^ Briefly, the mouse colon was opened longitudinally, and the fat and connective tissues were removed, washing out the fecal materials. After that, Kimwipe was used to clean and soak the extra water in the colon. The colon length and weight were measured to determine the colon thickness.

#### Assessment of disease activity index

During the DSS-induced colitis studies, a disease activity index (DAI) score was assessed to evaluate the progression of colitis as described previously.^8^ The DAI was a composite score determined by relative body weight loss, stool softness, and blood in mouse stool or rectum.

### Gut microbial composition analysis

Fecal contents were flash frozen, and bacterial DNA was extracted using the QIAamp DNA Stool Mini Kit (Qiagen Valencia, CA, USA) with a bead-beating step as previously described. Amplicon sequencing and detailed composition of the fecal microbiota using 16S rRNA bacterial gene sequencing were performed using the Illumina software at the University of Nebraska Medical Center’s Genomic Core facility. QIIME2 (Quantitative Insights into Microbial Ecology version 2, 2020.2) software with the DADA2 algorithm was used to determine the taxonomic diversity profiles of the microbiota in *Cldn3*KO and WT littermates. Sequences were denoised and clustered into Amplicon Sequencing Variants (ASVs) using the DADA2 algorithm. Taxonomic classification was performed using the QIIME2 workflow with the SILVA 132 reference set. For the microbiota-related analysis, the two genotypes, *Cldn3*KO and WT, were compared using the Wilcoxon t-test. A statistical difference of at least *P* < .05 was used to select the significant features between the genotypes. Alpha and beta diversity, relative frequency, and box plots were plotted using the R package.

### Fecal Microbial Transplantation (FMT)

To validate the role of gut dysbiosis in IBD following CLDN3 loss, FMT was performed using cecal microbial transplantation into germ-free mice. In brief, ceca with microbial content from *Cldn3*KO and WT littermates were snap-frozen. At the time of inoculum preparation, frozen ceca were introduced into an anaerobic chamber (Bactron600 Shel Lab, Cornelius, OR) to avoid oxygen exposure, and slurries were prepared by suspending cecal contents in pre-reduced sterile PBS with 10% glycerol (pH 7) at a dilution of 1:10. The cecal communities of both *Cldn3*KO and WT mice were transferred into germ-free C57BL/6J mice raised by the Nebraska Gnotobiotic Mouse Program at the University of Nebraska-Lincoln. After day 14 post-inoculation of cecal microbial content, acute DSS colitis was induced to study the effect of CLDN3 loss on microbial dysbiosis and colitis. At the end of the study, colon specimens were fixed in 10% formalin, embedded in paraffin, and subjected to H&E staining to evaluate histology and pathology. The degree of colitis pathology was assessed by pathologists at the University of Nebraska Medical Center, Omaha, in a blinded manner.

### Generation of pseudo-germ-free mice

To deplete the gut microbiota, *Cldn3*KO mice were treated with a cocktail of broad-spectrum antibiotics (ampicillin 1 g/L, neomycin sulfate 1 g/L, metronidazole 1 g/L, vancomycin 0.5 g/L) in drinking water for 2 weeks as previously described.^70^ The mice were monitored for adverse effects of the broad-spectrum antibiotic cocktail. After two weeks, fecal content was used to determine microbial depletion using standard bacterial inoculation on Luria broth agar culture plates. After confirming gut microbial depletion, the mice were subjected to DSS acute colitis.

### In vitro *stimulation of intestinal epithelial cells by dysbiotic fecal content*

To investigate the potential inflammatory responses of intestinal epithelial cells to gut dysbiosis, Caco-2 cells were challenged with fecal content from *Cldn3*KO and WT littermates. Briefly, *Cldn3*KO and WT mice were kept in separate cages for at least three weeks, after which fecal pellets were collected under sterile conditions. Equal amounts of fecal content were dissolved in phosphate-buffered saline and sonicated for 10 seconds at 20% amplitude. Following sonication, samples were divided into two parts, native and heat-inactivated for 10 minutes at 95°C respectively. 20 µl of native and heat-inactivated dysbiotic fecal content were added apically to the monolayer of intestinal epithelial cells for stated times points then cells were collected for immunoblot analysis.

### Measurements of trans-epithelial resistance

Cells were plated on transwell filters (0.4 μm). The confluent monolayer was treated apically with the fecal microbial content from both *Cldn3*KO and WT mice for 48 hrs. The trans-epithelial resistance was determined as described previously and is represented as Ω xcm2.^71^

### Immunoblot, immunofluorescence and immunohistochemistry analysis

Immunoblot, immunofluorescence, and immunohistochemistry analyses were performed as described previously.^71^

### RNA isolation for quantitative RT-PCR

Total RNA from mouse colon samples was extracted from 20 to 30 mg of tissue using the Quick-RNA Miniprep Kit (Zymo Rsearch, Irvine, CA) with DNase digestion. Total RNA (1ug) from each sample was reverse-transcribed using the iScript cDNA Synthesis Kit (Bio-Rad, Hercules, CA, USA). PCR was performed using a cytokine primer set (Supplementary Table S1). Each well-contained SYBR Green Master Mix (Bio-Rad), primer sets, and cDNA (30ng). Samples were loaded in duplicates in 96 well plates and run on a Bio-Rad CFX Connect (Bio-Rad, Hercules, CA, USA) real-time PCR machine. The Ct values were used to calculate the fold change. The normalization was performed using β-actin.

### GEO data collection, pre-processing and analysis of IBD patients

Whole-transcriptome data from mucosal biopsies of IBD patients were downloaded from the Gene Expression Omnibus (GEO) website (www.ncbi.nlm.nih.gov/geo/) using the GEO query package in R programming language. Publicly available datasets were used for the analysis (GSE53306, GSE111889, GSE48634, GSE65114, GSE16879, GSE36807, GSE13367, GSE14580, GSE38713, GSE75214, GSE59071, GSE9452, and GSE48958).^37,72–83^ All the data were batch normalized and log 2 transformed using ‘sva’ package in R bioinformatics software.^84,85^

### Statistical analysis

Statistical analyses were performed using data from at least triplicate experiments unless stated otherwise in the figure legends. Mean values with standard errors were used for independent Student’s t-tests or one-way ANOVA, and corrections for multiple comparisons were made using Tukey’s multiple comparison tests in Prism 9.5 (GraphPad Software Inc.). Statistical significance was set at *p* < .05.

## Supplementary Material

Supplementary Table.docx

## Data Availability

The data are available upon request. The data supporting the findings of this study are available from the corresponding author upon request.
