# Supplementary material for: Loss of claudin-3 expression increases colitis risk by promoting Gut Dysbiosis
Source: Gut Microbes. 2023 Nov 27;15(2):2282789. doi: 10.1080/19490976.2023.2282789 (PMC10730149; doi:10.1080/19490976.2023.2282789)
Supplement: Supplementary Table.docx [file KGMI_A_2282789_SM4301.docx]

**Supplemental Table 1 (Real-time PCR primers)**

| **Gene** | **Sense Primer** | **Antisense Primer** |
| --- | --- | --- |
| Actin | 5’-CCAGAGCAAGAGAGGTATCC-3’ | 5’-CTGTGGTGGTGAAGCTGTAG-3’ |
| TNF | 5’-CTGTGAAGGGAATGGGTGTT-3’ | 5’-GGTCACTGTCCCAGCATCTT-3’ |
| IL-6 | 5’-AGAGACTTCCATCCAGTTGC-3’ | 5’-TCCTTAGCCACTCCTTCTGT-3’ |
